# Divergent immunomodulatory and gut microbiota-modulating effects of *Sargassum* polysaccharides and oligosaccharides in delayed-type hypersensitivity mice

**DOI:** 10.1186/s40643-025-00948-8

**Published:** 2025-09-30

**Authors:** Yang-Ching Chen, Shih-Yuan Fang, Chien-Li Chen, Ming-Chih Fang, Yu-Ying Yang, Meng-Chou Lee, Chung-Hsiung Huang

**Affiliations:** 1 Department of Family Medicine, School of Medicine, College of Medicine, Taipei Medical University, Taipei, 110301 Taiwan; 2 Department of Family Medicine, Wan Fang Hospital, Taipei Medical University, Taipei, 116079 Taiwan; 3 School of Nutrition and Health Sciences, College of Nutrition, Taipei Medical University, Taipei, 110301 Taiwan; 4 Graduate Institute of Metabolism and Obesity Sciences, Taipei Medical University, Taipei, 110301 Taiwan; 5 Nutrition Research Center, Taipei Medical University Hospital, Taipei, 110301 Taiwan; 6 TMU Research Center for Digestive Medicine, Taipei Medical University, Taipei, 110301 Taiwan; 7 Department of Food Science, National Taiwan Ocean University, Keelung, 202301 Taiwan; 8 Department of Aquaculture, National Taiwan Ocean University, Keelung, 202301 Taiwan

**Keywords:** Delayed-type hypersensitivity, Gut microbiota, Oligosaccharides, Polysaccharides, *Sargassum*

## Abstract

Recent research has indicated that polysaccharides extracted from *Sargassum* (SP) possess promising activity in alleviating type I hypersensitivity reactions. However, effects of SP and *Sargassum* oligosaccharides (SO) on immune regulation and gut microbiota in type IV hypersensitivity remain unexplored. In this study, SP and SO were prepared and structurally characterized. SP contained high-molecular-weight fractions (866 kDa and 276 kDa), whereas SO was composed of low-molecular-weight components (3.74 kDa and 126 Da), lacked sulfate groups, and exhibited higher reducing sugar contents. The influence of SP and SO on immune regulation and the structure of gut microbial communities was examined using a mouse model of ovalbumin (OVA)-induced delayed-type hypersensitivity (DTH). Administration of SP and SO (25 or 250 mg/kg per day for 10 days) significantly attenuated DTH responses, evidenced by a decrease in footpad edema and a lower degree of cell infiltration. While both SP and SO had limited effects on serum IgG_1_ levels and splenic TGF-β production, treatment with SO at 250 mg/kg significantly reduced serum total IgG, OVA-specific IgG, and splenic IL-2 levels, while increasing IL-10 production. Notably, SO exerted the most pronounced effect in lowering IgG_2a_ and IFN-γ levels. Additionally, SO treatment led to distinct shifts in gut microbial profile, marked by elevated levels of *Muribaculaceae*, *Lactobacillus*, and *Bacteroides*. These microbial changes were accompanied by elevated concentrations of short-chain fatty acids (SCFAs). Collectively, these results indicate that SO holds potential as a functional dietary component for the alleviation of type IV hypersensitivity responses, through modulation of gut microbiota and immunomodulation.

**Graphical abstract:**

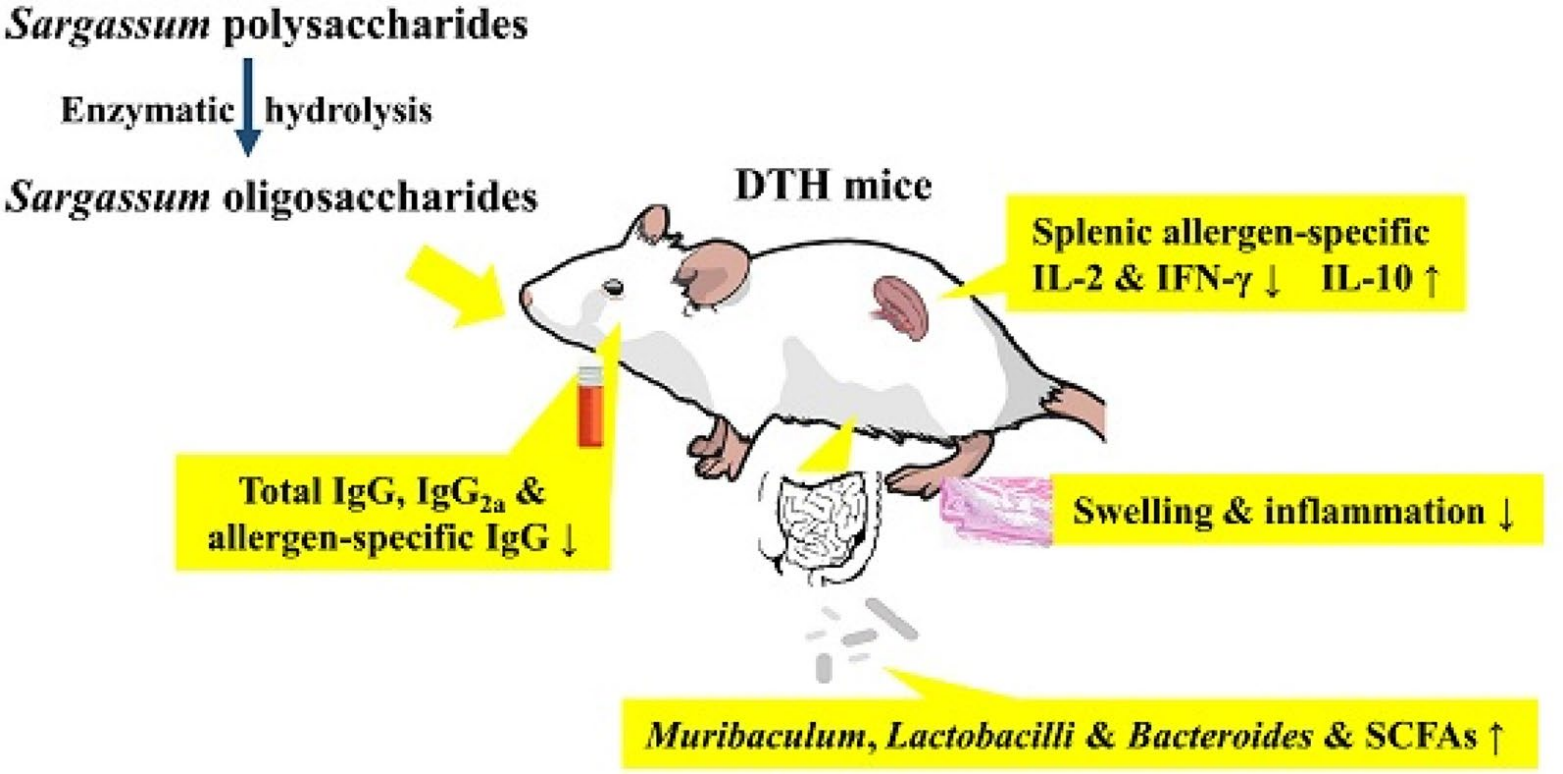

## Introduction

Algae are increasingly recognized as promising and sustainable food sources due to their rich nutritional profiles and relatively low environmental impact. Among them, *Sargassum*, brown macroalgae predominantly distributed across the western Pacific Ocean, is economically significant and have been traditionally utilized for habitat restoration, fertilizers, animal feed, functional foods, and herbal medicines (Rossignolo et al. 2022; Rushdi et al. 2020). Based on dry weight analysis, *Sargassum* comprises roughly 57% carbohydrates, 26% ash, 11% protein, 6% cellulose, and 0.5% lipids, underscoring its value as a potential source of bioactive constituents (Fan et al. 2017). Polysaccharides represent a major and biologically significant component of *Sargassum*. *Sargassum* polysaccharides (SP) have been shown to possess diverse biological activities (Byun 2015; Fan et al. 2017; Sun et al. 2018; Wen et al. 2016). *Sargassum* oligosaccharides (SO), derived through enzymatic hydrolysis of SP, have also demonstrated beneficial effects, including anti-diabetic and anti-inflammatory activities (Yang et al. 2019; Zhou et al. 2015). Despite these promising findings, comparative studies on the physiological functions of SP and SO, particularly in the context of immune disorders, remain limited. In particular, their potential effects against type IV hypersensitivity reactions have not been well characterized.

Delayed-type hypersensitivity (DTH), a form of type IV hypersensitivity, is a T cell-mediated immune response involving the recruitment of macrophages and other leukocytes to the site of antigen exposure. This represents the second most common form of allergic response, typified by inflammation of the affected tissues developing 24 to 48 h after allergen exposure (Kobayashi et al. 2001). T helper 1 (Th1) cells play a central role in mediating the DTH reaction by releasing cytokines such as IL-2 and IFN-γ, promoting lymphocytes and macrophages activation and inflammation (Black 1999). In contrast, the resolution of inflammation is supported by regulatory T (Treg) cells, which release cytokines like IL-10 and TGF-β (Palomares et al. 2014). Conventional treatment strategies, such as topical corticosteroids, often come with side effects, highlighting the need for safer and more natural therapeutic alternatives (Black 1999; Palomares et al. 2014).

Gut microbiota ferment dietary fibers to produce short-chain fatty acids (SCFAs), predominantly acetate, propionate, and butyrate, which are crucial regulators of immune function (Koh et al. 2016; Tan et al. 2014). These SCFAs modulate both innate and adaptive immunity, thereby guiding T cell subsets differentiation and cytokine secretion. In Th1-mediated immune responses, SCFAs suppress pro-inflammatory cytokines and promote IL-10 expression, while steering CD4⁺ T cells toward regulatory phenotypes to mitigate inflammation (Park et al. 2015; Sun et al. 2017). Accordingly, SCFAs-mediated modulation of gut-immune interactions may offer an approach to modulate Th1-driven hypersensitivity responses.

Recent studies have reported that crude *Sargassum* polysaccharides or hot water extracts can alleviate DTH-like responses in murine models of allergic contact dermatitis (Mitra et al. 2020; Woo et al. 2023). These findings suggest that *Sargassum*-derived components may modulate T cell-mediated immune reactions. To date, a direct comparison of the immunoprotective effects of SP and SO has not been conducted. Moreover, the contribution of gut microbiota and SCFAs production to these effects remains insufficiently explored. This research focused on evaluating and comparing the immune-modulating effects of SP and SO using a DTH model. Structural characterization of both SP and SO was performed, and their influences on allergen-specific immune responses, gut microbial communities, and SCFAs profiles were systematically examined to uncover their potential mechanisms of action.

## Materials and methods

### Materials

Chemicals were acquired from Sigma-Aldrich (St. Louis, MO, USA). Materials for cell culture procedures were supplied by GE Healthcare Life Sciences (Marlborough, MA, USA), while ELISA kits for antibody and cytokine detection were sourced from eBioscience, Inc. (San Diego, CA, USA). *Sargassum* were kindly supplied by Professor Meng-Chou Lee of the Department of Aquaculture, National Taiwan Ocean University, Keelung, Taiwan.

### Processing and characterization of SP and SO

The methods used for SP and SO production were adapted with slight modifications from prior research (Huang et al. 2022; Ou et al. 2023). 1 gram of *Sargassum* powder was dispersed in 1 L of deionized water and subjected to autoclaving at 121 °C for 20 min. The mixture was then centrifuged, and the resulting supernatant was mixed with 95% ethanol to induce precipitation. The resulting solid was redissolved in 1 L deionized water and desalted by electrodialysis (Micro Acilyzer S3, Astom, Tokyo, Japan) using 0.1 M Na₂SO₄ as the electrolyte at 18.3 V for 30 min. This solution was subsequently freeze-dried to yield SP powder. For the preparation of SO, a 2% (w/v) solution of SP in PBS was enzymatically digested using Celluase at 37 °C for 48 h. The enzymatic reaction was terminated by autoclaving the mixture for 20 min. The hydrolysate was then processed using an ultrafiltration membrane to isolate oligosaccharide fractions smaller than 3 kDa. The final SO product was obtained by freeze-drying the filtered solution for further use.

Analyses of total phenolics, sugars (total and reducing), peptides, sulfate groups, molecular weights of SP and SO, and their functional groups were performed based on procedures reported in previous literature (Huang et al. 2022; Wei et al. 2022). Briefly, total phenolic content was determined by reacting 50 µL of sample with Folin–Ciocalteu reagent and Na₂CO₃, followed by incubation in the dark and measurement of absorbance at 735 nm. Total sugar was measured by mixing diluted samples with phenol and concentrated sulfuric acid, reacting at room temperature, and reading absorbance at 480 nm using glucose as the standard. Reducing sugar was quantified by reacting the sample with 3,5-dinitrosalicylic acid reagent, heating, then measuring absorbance at 546 nm after cooling. Peptide content was determined by reacting the sample with o-phthaldialdehyde reagent and measuring absorbance at 340 nm, using leucine-glycine as the standard. Sulfate groups were quantified by hydrolyzing samples in HCl, reacting with BaCl₂-gelatin reagent, and measuring absorbance at 360 nm using Na₂SO₄ standards. Molecular weight was determined by HPLC with Shodex Asahipak SB-804 HQ (7.5 × 300 mm) and Shodex Asahipak GS-320 HQ (7.5 × 300 mm) columns using deionized water containing 0.02% NaN₃ as the mobile phase; detection was performed by refractive index, and molecular weights were calculated from standard curves. FTIR spectra (Tensor 27, Bruker Optics, Ettlingen, Bruker, Germany) were recorded by mixing 1 mg of dry sample with 99 mg KBr, pressing into pellets, and scanning in the range of 4000–400 cm⁻¹.

### Animal treatment and DTH induction

All animal procedures adhered to the National Research Council’s Guide for the Care and Use of Laboratory Animals and received approval from the NTOU Institutional Animal Care and Use Committee (NTOU IACUC-108040). Five-week-old female BALB/c mice, obtained from the National Laboratory Animal Center of Taiwan, were acclimatized for seven days prior to the experiment. Housing conditions included a temperature of 24 ± 2 °C, humidity of 55 ± 20%, and a 12-h light/dark cycle. Mice had free access to sterile distilled water and LabDiet 5001 chow (PMI Nutrition International, Arden Hills, MN, USA). The mice were then allocated into six groups of five animals each: naïve (NA), vehicle (VH), and treatment groups. The animal experiments were independently repeated three times to ensure reproducibility and reliability of the results. The VH group was given 0.1 mL of phosphate-buffered saline (PBS) daily via oral gavage, whereas the treatment groups received SP or SO at doses of 25 mg/kg (SPL or SOL) or 250 mg/kg (SPH or SOH) suspended in 0.1 mL of PBS. A positive control was not included, as no standard immunosuppressive agent has been well validated for use in the employed DTH model. DTH induction followed a previously described protocol with minor modification. Except for the NA group, all mice were sensitized on day 3 by intraperitoneal injection of 0.1 mL PBS containing 100 µg of OVA and 2 mg of aluminum hydroxide. On day 9, each animal received a subcutaneous injection of 200 µg OVA in 20 µL PBS into the footpad (Allen 2013). Footpad swelling, as a measure of DTH response, was recorded before and 24 h after challenge. Fresh fecal samples were collected for microbiota and SCFA analysis. Serum was collected, and mice were humane euthanized through inhalation of CO₂, in accordance with ethical guidelines, to harvest footpad tissues. The tissues were fixed in 10% neutral buffered formalin and sent to the National Laboratory Animal Center (Taipei, Taiwan) for paraffin embedding, sectioning at 5 μm thickness, and staining with hematoxylin and eosin (H&E) using standard protocols (Ou et al. 2024). The stained sections were then observed under an Olympus BX53 microscope to evaluate inflammation induced by OVA and the effects of treatment. Spleens were also collected to prepare splenocyte suspensions (Wei et al. 2022).

### Detection of OVA-stimulated cytokine secretion from splenocytes and IgG production in serum

Splenocytes isolated from each mouse were plated at 6 × 10⁶ cells/mL in 24-well plates and cultured with OVA (50 µg/mL) for 24 to 72 h. Culture supernatants were gathered to measure IL-2, IFN-γ, IL-4, IL-5, IL-10, and TGF-β levels by ELISA (Wei et al. 2022). Serum samples were also analyzed for total IgG, IgG_1_, IgG_2a_, and OVA-specific IgG using the same method, following the manufacturer’s instructions. Briefly, 96-well plates were coated with ELISA coating buffer and incubated overnight at 4 °C. After washing, wells were blocked with blocking buffer for 1 h at room temperature, then incubated with serially diluted standards, serum, or supernatant samples for 1 h. Following another wash, detection antibody was added and incubated for 1 h. After washing, HRP solution was added to each well and incubated at room temperature for 30 min. The wells were then washed, developed with TMB substrate for 15 min, and the reaction was stopped with 6 N H₂SO₄. Absorbance at 450 nm was measured using a microplate reader (Synergy HT, BioTek Instruments, Winooski, VT, USA), and concentrations were calculated using standard curves.

### Fecal microbiota analysis

Fecal DNA extraction and 16 S rRNA sequencing followed a previously established protocol (Ou et al. 2023). Using the QIAamp PowerFecal DNA Kit (Qiagen), genomic DNA was extracted and then standardized to a concentration of 1 ng/µL. The entire 16 S rRNA gene was amplified via PCR using primers tagged with barcodes, performing 20 to 27 cycles. Amplicons were checked by gel electrophoresis, purified with AMPure PB beads, and used to build SMRTbell libraries per PacBio guidelines. Sequencing was performed on the PacBio Sequel IIe platform to generate high-fidelity reads. Data processing involved DADA2 for filtering and chimera removal, while QIIME 2 handled taxonomic assignment.

### Fecal microbiota analysis

Fresh fecal sample (50 mg) from each mouse was combined with 20 µL of 1000 ppm 2-ethylbutyric acid (internal standard) and 500 µL saturated NaCl solution, then chilled on ice for. The sample was homogenized for 3 min, then 20 µL of 10% sulfuric acid was added and brief vortexing. SCFAs were isolated using 800 µL of diethyl ether, followed by centrifugation at 12,000 rpm for 15 min at 4 °C. The organic phase was then dried over 0.25 g of anhydrous Na₂SO₄ and filtered through a 0.22 μm membrane. The analysis was conducted using gas chromatography equipped with a flame ionization detector (GC-FID) on an Agilent 8860 system using a NUKOL column (30 m × 0.25 mm × 0.25 μm). The oven temperature was initially set to 100 °C for 1 min, then ramped up to 150 °C at a rate of 5 °C per min and maintained for 5 min, total 16 min. The injector and detector temperatures were set at 270 °C and 280 °C, respectively, using nitrogen as the carrier gas at a flow rate of 2.0 mL/min. A 2 µL sample was injected in split mode with a ratio of 20:1. Quantification was performed using standard curves (0–100 ppm) of acetic, propionic, and butyric acids, with 2-ethylbutyric acid as the internal standard. SCFAs were identified by retention times and quantified using the internal standard method, expressed as mmol/kg feces.

### Statistical analysis

Comparisons between groups were performed using ANOVA, while differences between the test and control groups were analyzed using Tukey’s test (SigmaPlot V14, Systat Soft-ware Inc.). Statistical significance was defined as *p* < 0.05.

## Results

### Characterization of SP and SO

**Fig. 1:**
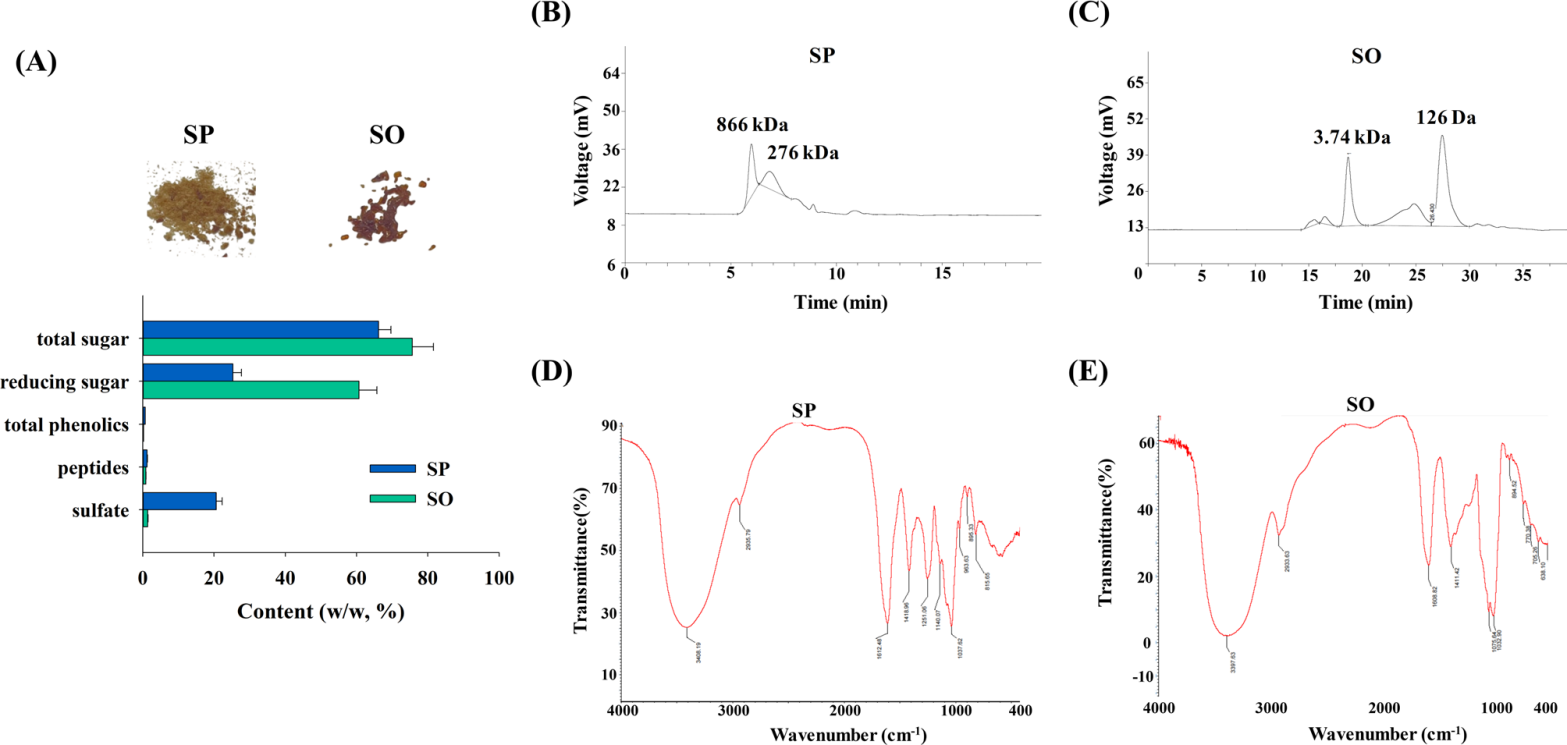
Characterization of SP and SO. **A** The morphology, composition, **B**, **C** HPLC and **D**, **E** FTIR spectra of SP and SO

After lyophilization, morphology of SP and SO powder was distinct. The color of SP was light brown, and that of SO was dark brown (Fig. 1A). In SP, total sugars, reducing sugars, and sulfate were identified as the major components. In comparison, SO showed elevated levels of reducing sugars, while the sulfate content was nearly eliminated. Both SP and SO contained only trace amounts of total phenolics and peptides (Fig. 1A). The molecular weights of SP and SO were assessed and calibrated against standard curves generated from known polysaccharides and oligosaccharides. The SP chromatogram showed two main peaks at approximately 866 kDa and 276 kDa (Fig. 1B), while SO displayed major peaks near 3.74 kDa and 126 Da (Fig. 1C). FTIR analysis revealed shared characteristic peaks for both SP and SO, including broad O–H stretching at 3347–3408 cm⁻¹, C–H vibrations at 2924–2293 cm⁻¹, carboxylate (C=O) stretches at 1608–1612 cm⁻¹, alkyl bending at 1411–1425 cm⁻¹, and glycosidic bond (C–O–C) stretching at 1032–1037 cm⁻¹. Notably, SP showed unique sulfate group (S = O) peaks at 1251 and 1140 cm⁻¹, whereas SO exhibited distinct C–H and C–S stretching vibrations at 1411 and 638 cm⁻¹, respectively (Fig. 1D and E). Accordingly, the employed enzyme could effectively hydrolyze SP, and the enzymatic hydrolysis may result in the removal of sulfate groups.

### SP and SO attenuated DTH-induced footpad swelling and inflammation

**Fig. 2:**
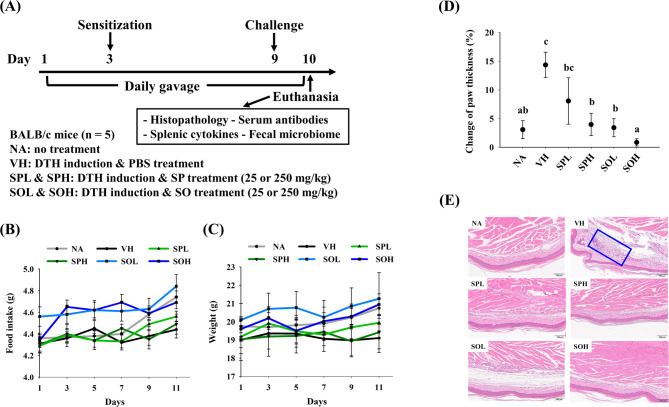
Impact of SP and SO on DTH response. **A** Experimental groups and DTH induction protocol in BALB/c mice. **B** Food intake and **C** Changes in body weight of mice in each group throughout the experiment. **D** Footpad swelling as a measure of DTH assessed by comparing thickness before and 24 h after the challenge. **E** Representative images of footpad tissues, and blue box indicate cell infiltration. Results are expressed as mean ± SEM (*n* = 5) and reflect one of three independent experiments that consistently demonstrated similar trends. Different letters (a–c) indicate significant differences (*p* < 0.05) among groups

DTH responses in OVA-sensitized mice typically appear 24 h after OVA footpad injection, presenting as swelling and infiltration of inflammatory cells (Fig. 2A) (Black 1999). Throughout the experimental period, mice in all groups maintained stable food intake and body weight (Fig. 2B and C). Aside from footpad swelling associated with the DTH response, no abnormal behaviors or signs of distress were observed, suggesting that SP and SO at the administered doses did not produce noticeable adverse effects. As shown in Fig. 2D, mice of the VH group exhibited a significant rise in footpad swelling relative to that in the NA group, indicating a strong inflammatory response following OVA challenge. In contrast, the treatment groups, particularly the SOH group, showed markedly reduced footpad swelling, suggesting that administration of SP or SO effectively attenuated footpad swelling associated with DTH. H&E-stained sections from the NA and treatment groups showed minimal cell infiltration at the footpad, while the VH group displayed extensive infiltration. Histopathological analysis of footpad tissues revealed a reduced local inflammatory response in the treatment groups, demonstrating the ability of SP and SO to mitigate DTH (Fig. 2E).

### Selective modulation of serum antibodies by SP and SO

**Fig. 3:**
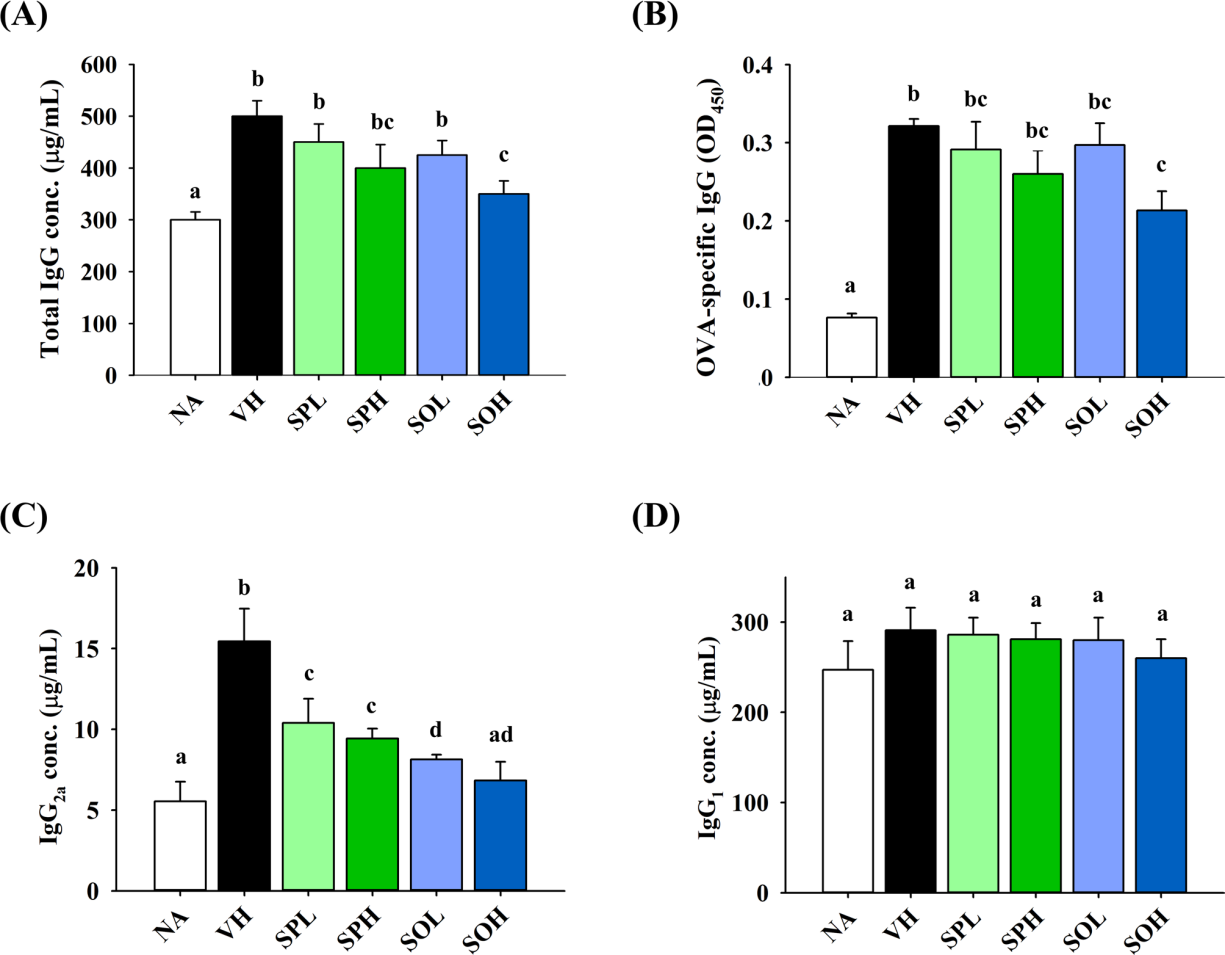
Influence of SP and SO on serum antibody levels in DTH mice. Prior to euthanasia, serum was collected individually from each mouse. Subsequently, ELISA was used to measure serum concentrations of **A** total IgG, **B** OVA-specific IgG, **C** IgG_2a_, and **D** IgG_1_. Results are expressed as mean ± SEM (*n* = 5) and reflect one of three independent experiments that consistently demonstrated similar trends. Different letters (a–d) indicate significant differences (*p* < 0.05) among groups

To evaluate the effects of SP and SO on humoral immune responses, serum concentrations of IgG and its subclasses were measured. The VH group exhibited markedly elevated total IgG, OVA-specific IgG, and IgG_2a_ concentrations compared to the NA group (Fig. 3A–C), indicating an amplified immune response following OVA sensitization and challenge. Notably, total IgG and OVA-specific IgG levels were similar in both the VH group and treatment groups, with the exception of SOH group, which showed a marked reduction in both antibodies (Fig. 3A and B). IgG_2a_ levels were significantly decreased in SPH, SOL and SOH groups, suggesting a downregulation of Th1-associated antibody production (Fig. 3C). In contrast, IgG_1_ concentrations remained consistent across all experimental groups (Fig. 3D), indicating a selective effect on the Th1-related IgG_2a_ isotype rather than a broad suppression of antibody production.

### Impact of SP and SO on Splenic T cell cytokine profiles

**Fig. 4:**
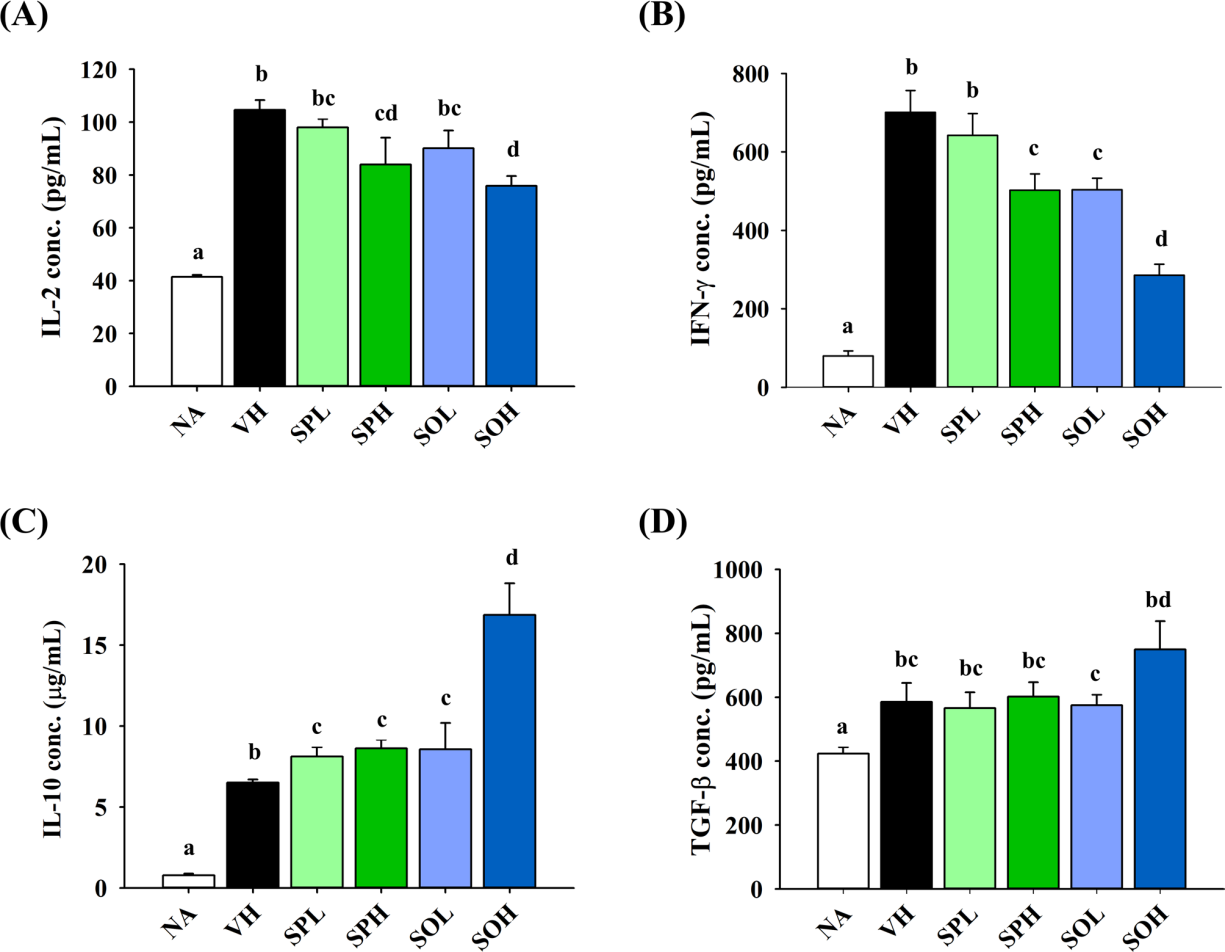
Influence of SP and SO on cytokine production. Splenocytes were isolated from each mouse and prepared for incubation with OVA. After incubation, supernatants were collected to quantify cytokine levels, specifically **A** IL-2, **B** IFN-γ, **C** IL-10, and **D** TGF-β. Results are expressed as mean ± SEM (*n* = 5) and reflect one of three independent experiments that consistently demonstrated similar trends. Different letters (a–d) indicate significant differences (*p* < 0.05) among groups

Given the potential of SP and SO to suppress Th1-skewed responses, splenic cytokines linked to Th1, Th2, and Treg cells were analyzed to assess allergen-specific immune modulation. The VH group exhibited a marked rise in measurable cytokine levels, indicating effective activation of allergen-specific immune responses (Fig. 4A–D). IL-2 concentrations were generally consistent among the treatment groups relative to the VH group, except for a notable reduction observed in the SOH group (Fig. 4A). In contrast, IFN-γ levels were significantly reduced in SPH, SOL and SOH groups, suggesting suppression of the Th1-dominant immune response (Fig. 4B). Moreover, SO at 250 mg/kg upregulated the production of IL-10 (Fig. 4C). However, neither SP nor SO treatment alter the production of TGF-β (Fig. D). To assess the impact of SP and SO on Th1/Th2 immune regulation, the level of IL-4, a signature cytokine produced by Th2 cells, was measured. However, IL-4 and IL-5 concentrations fell below the measurable range. This outcome is not unexpected, as the DTH model employed in this study is characterized by a predominantly Th1-driven immune response. In such models, Th1 cytokines are typically elevated, whereas Th2 cytokines are suppressed. The absence of detectable IL-4 and IL-5 further supports the Th1-skewed immune environment induced by OVA sensitization and challenge in this experimental setup.

### Modulation of gut microbiota and SCFAs profiles by SP and SO intake

**Fig. 5:**
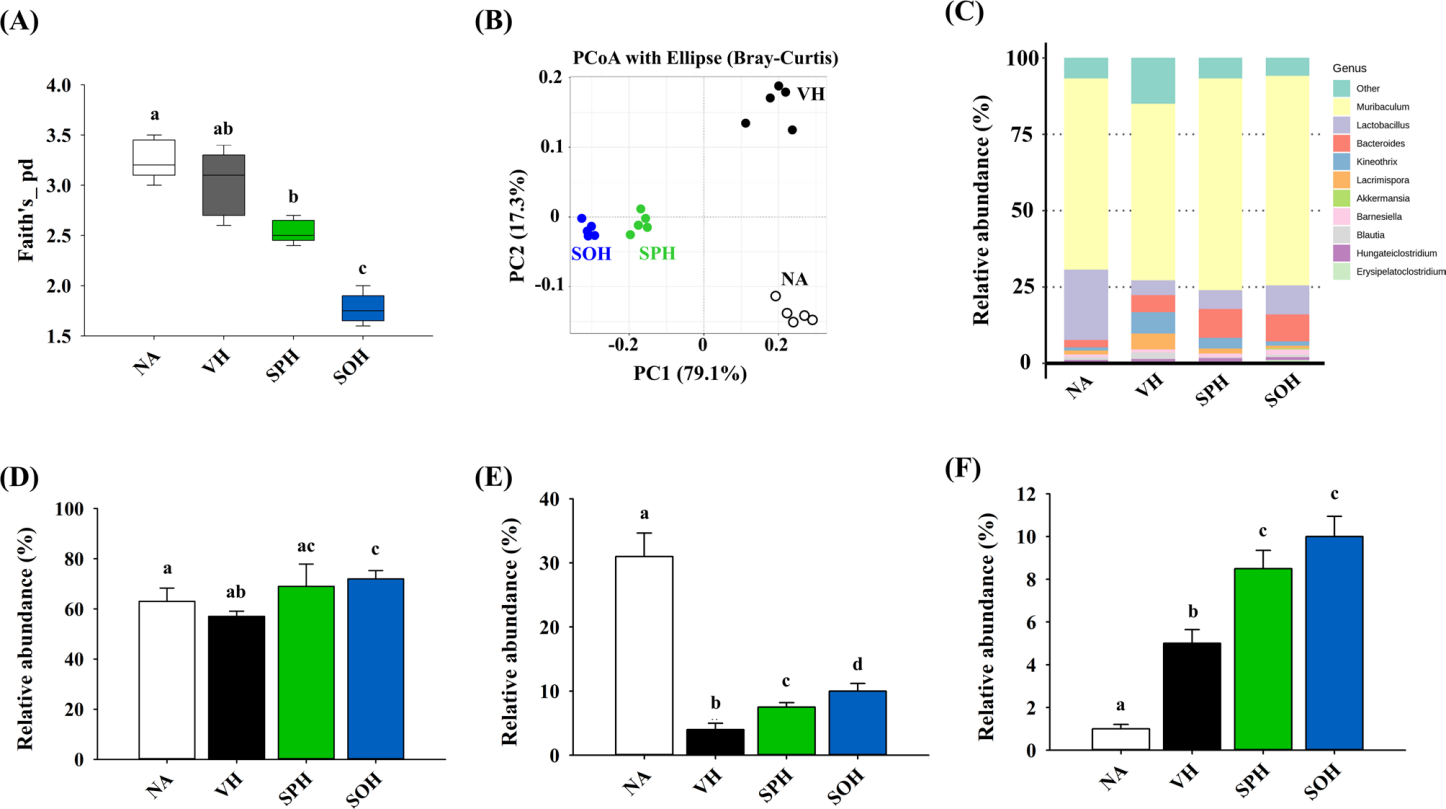
Impact of SP and SO treatment on gut microbiota. Fresh fecal samples were collected post-euthanasia from each mouse, and DNA was extracted for full-length 16 S rRNA gene sequencing. The results include**A** Faith’s phylogenetic diversity (PD) of α-diversity, **B** principal coordinates analysis (PCoA) of β-diversity, **C** genus-level microbial composition, and the relative abundances of **D** *Muribaculam*, **E** *Lactobacillus*, and (F) *Bacteroides*. Results are expressed as mean ± SEM (*n* = 5) and reflect one of three independent experiments that consistently demonstrated similar trends. Different letters (a-d) indicate significant differences (*p* < 0.05) among groups

**Fig. 6:**
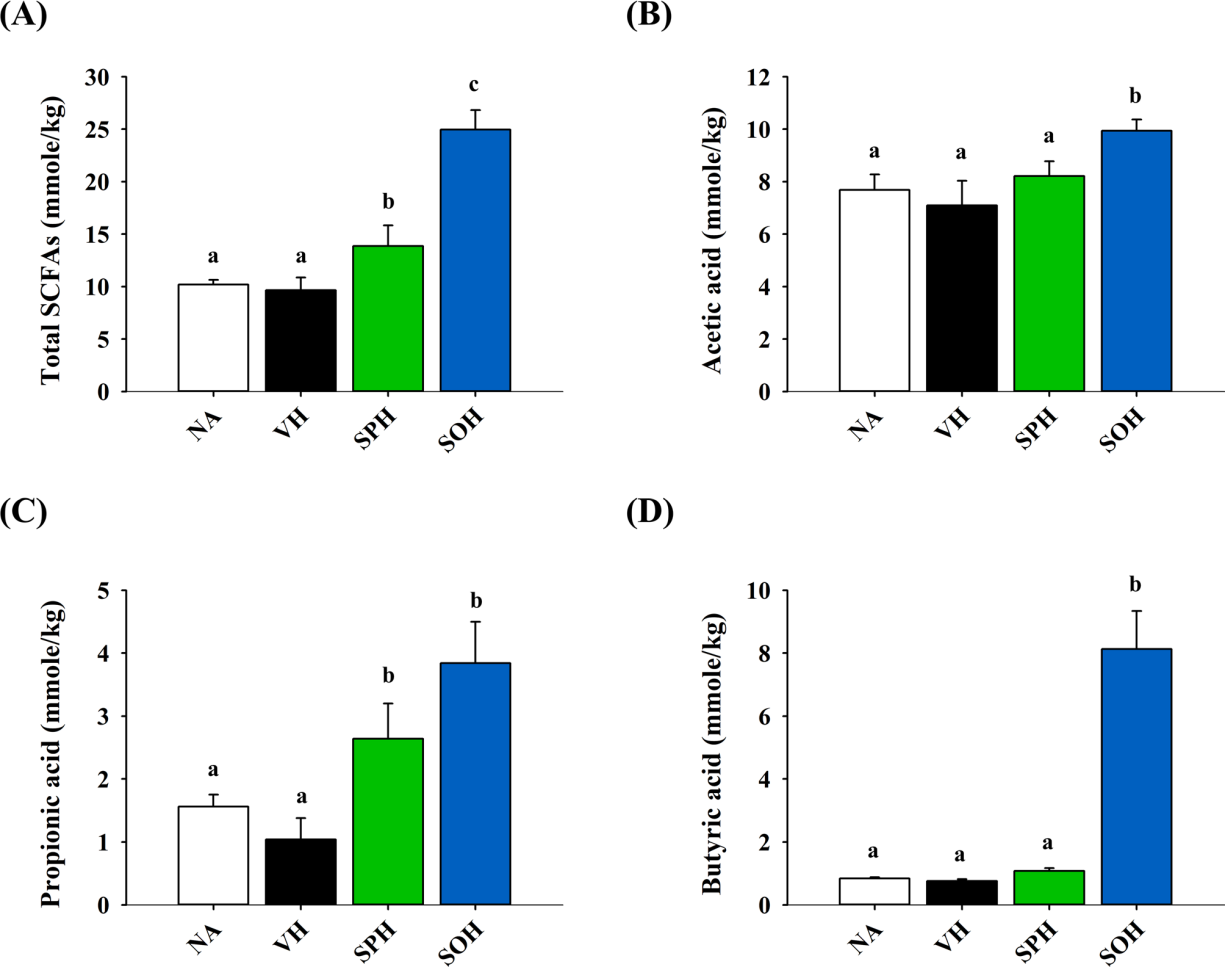
Impact of SP and SO treatment on gut SCFAs. Fresh fecal samples were collected post-euthanasia from each mouse, and SCFAs were extracted for analysis of **A** total SCFAs, **B** acetic acid, **C** propionic acid and **D** butyric acid using GC-FID. Results are expressed as mean ± SEM (*n* = 5) and reflect one of three independent experiments that consistently demonstrated similar trends. Different letters (a–c) indicate significant differences (*p* < 0.05) among groups

To assess how SP and SO supplementation influences gut microbial ecology, we conducted 16 S rDNA sequencing using fecal samples. Given the recognized role of intestinal microbiota in maintaining immune equilibrium and influencing hypersensitivity responses, we focused on microbial community structure and associated SCFAs production. Relative to the VH group, a significant reduction in Faith’s phylogenetic diversity, a common alpha-diversity metric, was observed exclusively in the SOH group, while the SPH group showed no such change (Fig. 5A). Beta-diversity analysis using principal coordinates analysis (PCoA) revealed consistent microbial profiles within each treatment group, along with distinct separation between groups (Fig. 5B). Compared to the NA group, the VH group had decreased Lactobacillus and increased *Muribaculum* abundance. The DTH-associated microbial shifts were partially reversed by SPH treatment and more markedly restored by SOH intervention, as indicated by increased levels of *Muribaculum*, *Lactobacillus*, and *Bacteroides* (Fig. 5C–F). Because SCFAs derived from gut microbiota play key roles in immunomodulation, fecal SCFA levels were further quantified. Although no notable differences were found between the NA and VH groups, treatment with SOH resulted in a significant elevation of total SCFA levels, encompassing acetic, propionic, and butyric acids (Fig. 6A–D).

## Discussion

The main constituents of *Sargassum* are carbohydrates, especially polysaccharides and oligosaccharides, known for their immune-regulating and anti-inflammatory effects (Byun 2015; Rushdi et al. 2020; Wen et al. 2016). The presence of sulfate groups is considered key to enhancing the bioactivity of these algal polysaccharides (Kazłowski et al. 2012). In this study, the distinctive sulfate group was detected in SP but absent in SO. Thus, the employed extraction process likely retained most sulfated polysaccharides from *Sargassum*, while enzymatic hydrolysis may have resulted in sulfate group loss in SO. Given that SO was more effective than SP in reducing DTH, molecular weight may play a key role in immunomodulatory activity, suggesting distinct mechanisms of action. Consequently, humoral and cellular immune responses were further investigated.

Immunoglobulin isotype profiles are shaped by the Th1/Th2 balance. IL-4–driven Th2 responses typically increase the IgG_1_/IgG_2a_ ratio, while IFN-γ–mediated Th1 responses are associated with a higher IgG_2a_/IgG_1_ ratio (Coffman et al. 1993). Serological results suggest that SP and SO may promote a shift from Th1 to Th2 dominance. To explore this, cytokine levels related to T cell subsets were measured. The ability of SP and SO to modulate the immune response was demonstrated by their inhibition of Th1-related markers like IgG_2a_, IL-2, and IFN-γ. The effects of *Sargassum* extracts are thought to involve maintaining immune tolerance, as indicated by elevated IL-10 levels (Burrello et al. 2018). Although TGF-β production was not significantly altered in treated mice, IL-10 levels were notably elevated in the SOH group, further indicating that immune tolerance may be a key mechanism by which SO mitigates DTH responses.

To date, limited research has explored the effects of algal components on DTH, with findings remaining inconsistent. For example, polysaccharides from *Petalonia binghamiae* and *Spirulina pacifica* have been shown to suppress inflammatory cytokine generation via TLR4, thereby alleviating DTH responses (Tominaga et al. 2010). In a rat DTH model, *Eucheuma cottonii* extract at doses of 150 and 300 mg/kg mitigated footpad inflammation (Anyanji et al. 2015). Earlier research demonstrated that *Porphyra* polysaccharides can reduce serum IgE levels in rodent models of DTH (Cao et al. 2016; Ishihara et al. 2005). Conversely, *Chlorella vulgaris* protein hydrolysates (500 mg/kg) were found to enhance DTH response recovery (Morris et al. 2007). Although numerous studies have reported the immunomodulatory effects of *Sargassum* in aquatic organisms, only a few in vivo studies investigated the anti-allergic or immunomodulatory potentials of *Sargassum* on mammals (Lee et al. 2020; Liyanage et al. 2022; Sönmez et al. 2021; Victoriano-Blancia et al. 2022). In mice, administration of ethanol extract of *S. horneri* (62.5 and 125 µg per mouse) decreased passive cutaneous anaphylaxis reaction (Han et al. 2020a). In another study, ethanol extract of *S. horneri* treatment (10–100 mg/kg) also attenuated atopic dermatitis, suppressed serum levels of IgG_1_ and IgG_2a_ and decreased atopic dermatitis-related cytokine production (Han et al. 2020b). However, the daily administration of *S. horneri* at 50 and 100 mg/kg exhibited a distinct effect. It led to a significant rise in the spleen and thymus weights, an increased count of blood immune cells, enhanced expression of Th1 cytokines, and a notable boost in natural killer cell activity among mice with cyclophosphamide-induced immunosuppression (Kim et al. 2022). The differences in immunomodulatory effects observed in the above studies may be attributed to variations in the employed algal species, extraction methods, and the animal models.

Our findings suggest a triad interplay between *Muribaculum*, *Lactobacillus*, and *Bacteroides* in enhancing SCFAs production and calibrating Th1-driven DTH. *Muribaculum*, a prominent SCFA producer, increases propionate and acetate levels that act via GPR43 on Th1 cells to enhance IL-10 while preserving IFN-γ, thereby limiting immune pathology (Burrello et al. 2018). *Bacteroides* contributes both SCFAs and immunomodulatory polysaccharide A, which activates TLR2 on CD4⁺ T cells, elevating T-bet/STAT4 signaling, IFN-γ secretion, and IL-10 production (Troy and Kasper 2010; Zhang et al. 2023). Although *Lactobacillus* primarily produces lactate, it engages in cross-feeding with butyrate-producing microbes, boosting butyrate, a potent SCFA that reinforces epithelial integrity, inhibits histone deacetylases, and skews T cells toward IL-10-producing, T-bet–expressing phenotypes (Hiippala et al. 2018). SCFAs such as propionate and butyrate thus appear to fine-tune Th1 responses, optimizing IFN-γ production necessary for effective DTH while inducing IL-10 to mitigate excessive inflammation (Burrello et al. 2018). The concurrent expansion of *Muribaculum*, *Lactobacillus*, and *Bacteroides* in our model aligns with improved DTH profiles, supporting a mechanism where microbial metabolism shapes protective yet regulated immunity. Further work is needed to confirm causality, particularly using germ-free or mono-colonized mice and SCFA receptor knockouts. Nevertheless, our data highlight a promising microbiome-based approach to modulate Th1 responses and manage DTH-related conditions via targeted microbial and metabolic interventions.

## Conclusion

This study demonstrates that both SP and SO can attenuate DTH responses in mice, with SO exhibiting more pronounced immunomodulatory effects, potentially due to its lower molecular weights. SO effectively inhibited Th1-related cytokines and antibodies, while favorably altering gut microbiota and boosting SCFAs levels. This study is the first to directly compare the immunomodulatory properties of SP and SO and to investigate gut microbiota’s role in DTH. The results highlight the potential of SO as a functional dietary agent for managing type IV hypersensitivity-related disorders via microbiota-driven immune modulation.

## Data Availability

Data will be made available on request.
